# Case Report: transient migratory pulmonary nodules with bronchial artery malformations in an autosomal dominant polycystic kidney disease patient: a case of recurrent hemoptysis

**DOI:** 10.3389/fmed.2026.1949035

**Published:** 2026-10-14

**Authors:** Yi Hu, Jie Zhang, Ting Wang, Bing Xue

**Affiliations:** Department of Respiratory and Critical Care Medicine, Chuiyangliu Hospital Affiliated with Tsinghua University, Beijing, China

**Keywords:** autosomal dominant polycystic kidney disease, bronchial artery malformation, hemoptysis, polycystic kidney, pulmonary nodule

## Abstract

Autosomal dominant polycystic kidney disease (ADPKD) is associated with vascular malformations, but bronchial artery involvement with migratory pulmonary nodules has not been reported. We describe an 81-year-old man with recurrent hemoptysis and migratory nodules on chest CT. Digital subtraction angiography revealed tortuous, dilated bronchial arteries; embolization controlled bleeding and nodules resolved. He had polycystic kidney and liver disease, a prior intracranial aneurysm, and a PKD1 missense variant (c.8426C > T). Common causes of hemoptysis were excluded. This case describes co-occurrence of bronchial artery malformation, recurrent hemoptysis and migratory pulmonary nodules in a patient with genetically confirmed ADPKD, raising the possibility that bronchial arterial involvement could be a rare, under-recognized vascular manifestation associated with ADPKD.

## Introduction

Autosomal dominant polycystic kidney disease (ADPKD) is the most common monogenic kidney disorder and the fourth leading cause of kidney failure worldwide (1). Extra-renal cysts commonly occur in the liver (90%), pancreas (19%), and seminal vesicles (40%) (1–3). ADPKD is also associated with vascular malformations, including intracranial aneurysms (10%–25%) and, more rarely, aneurysms and dissections of the aortic root, thoracic aorta, and coronary arteries (4). Regarding respiratory comorbidities, the prevalence of bronchiectasis and chronic obstructive pulmonary disease (COPD) is increased in patients with ADPKD, affecting up to 37% and 30% of patients, respectively (5, 6). To date, no studies have reported an association between ADPKD and migratory pulmonary nodules or bronchial artery malformation. We report a case of a patient who presented with recurrent hemoptysis and migratory pulmonary nodules, in whom subsequent investigation revealed concurrent bronchial artery malformation and ADPKD.

## Case report

An 81-year-old male was admitted with a chief complaint of “intermittent hemoptysis for 9 years, recurring for 5 days.” The patient first developed episodes of mild, intermittent hemoptysis in 2016, which resolved spontaneously without investigation or treatment. In July 2018, a recurrence of hemoptysis led to a chest CT scan, which revealed a nodule in the left lower lobe. Subsequent CT scans demonstrated migratory pulmonary nodules (Figure 1), which resolved with supportive care including hemostatic therapy. During this period, he also developed a spontaneous left-sided pneumothorax that resolved following closed thoracic drainage, most likely secondary to his COPD and emphysema.

**Figure 1:**
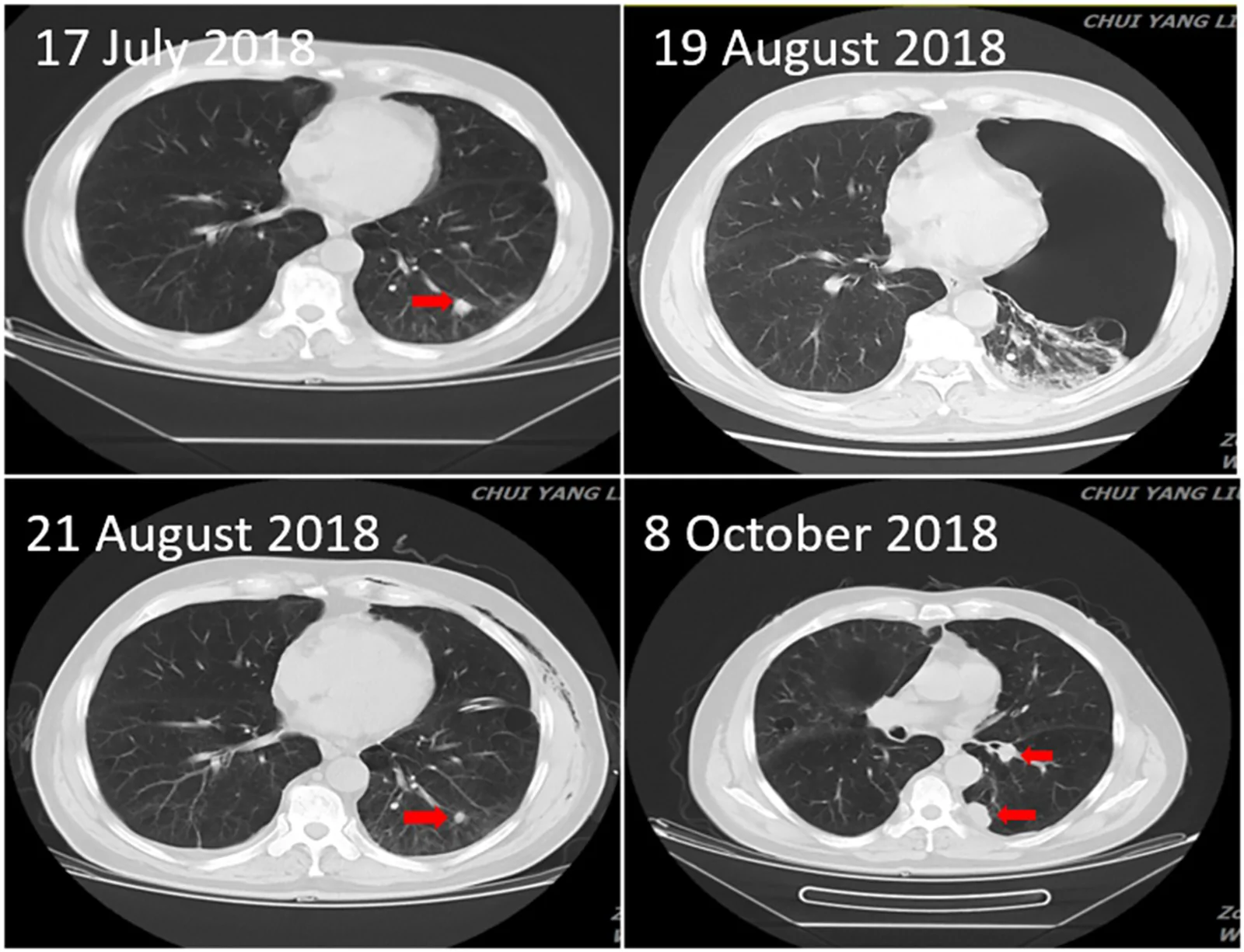
Serial chest CT images showing migratory pulmonary nodules in 2018. Solid red arrows indicate active pulmonary nodules.

In February 2024, hemoptysis recurred. Chest CT showed new nodules in the dorsal segment of the left lower lobe. Bronchoscopy revealed a small amount of fresh blood at the opening of the left lower lobe bronchus; bronchoalveolar lavage fluid (BALF) analysis was negative for pathogens. A vascular surgery consultation suggested a bronchial artery malformation, and digital subtraction angiography (DSA) revealed tortuous bilateral bronchial arteries with significantly increased diameters (approximately 2 mm). Embolization of the tortuous bilateral bronchial arteries was performed, which successfully stopped the hemoptysis (Figure 2). A follow-up chest CT obtained 2 months after embolization showed resolution of the nodule in the dorsal segment of the left lower lobe (Figure 3).

**Figure 2:**
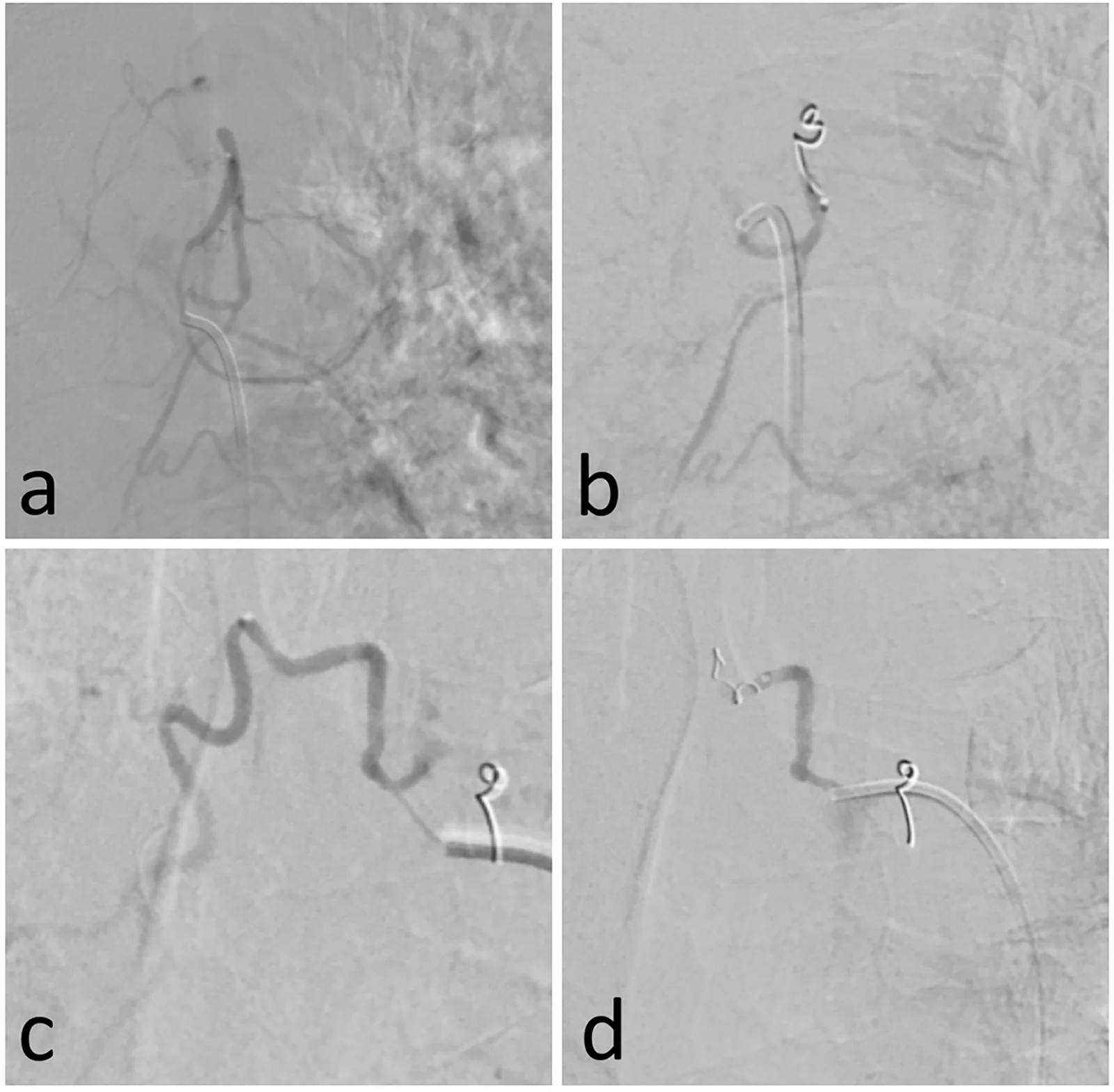
Intraoperative bronchial arteriography during bronchial artery embolization. **a** and **b** show the right malformed bronchial artery before and after embolization, respectively. **c** and **d** show the left malformed bronchial artery before and after embolization, respectively.

**Figure 3:**
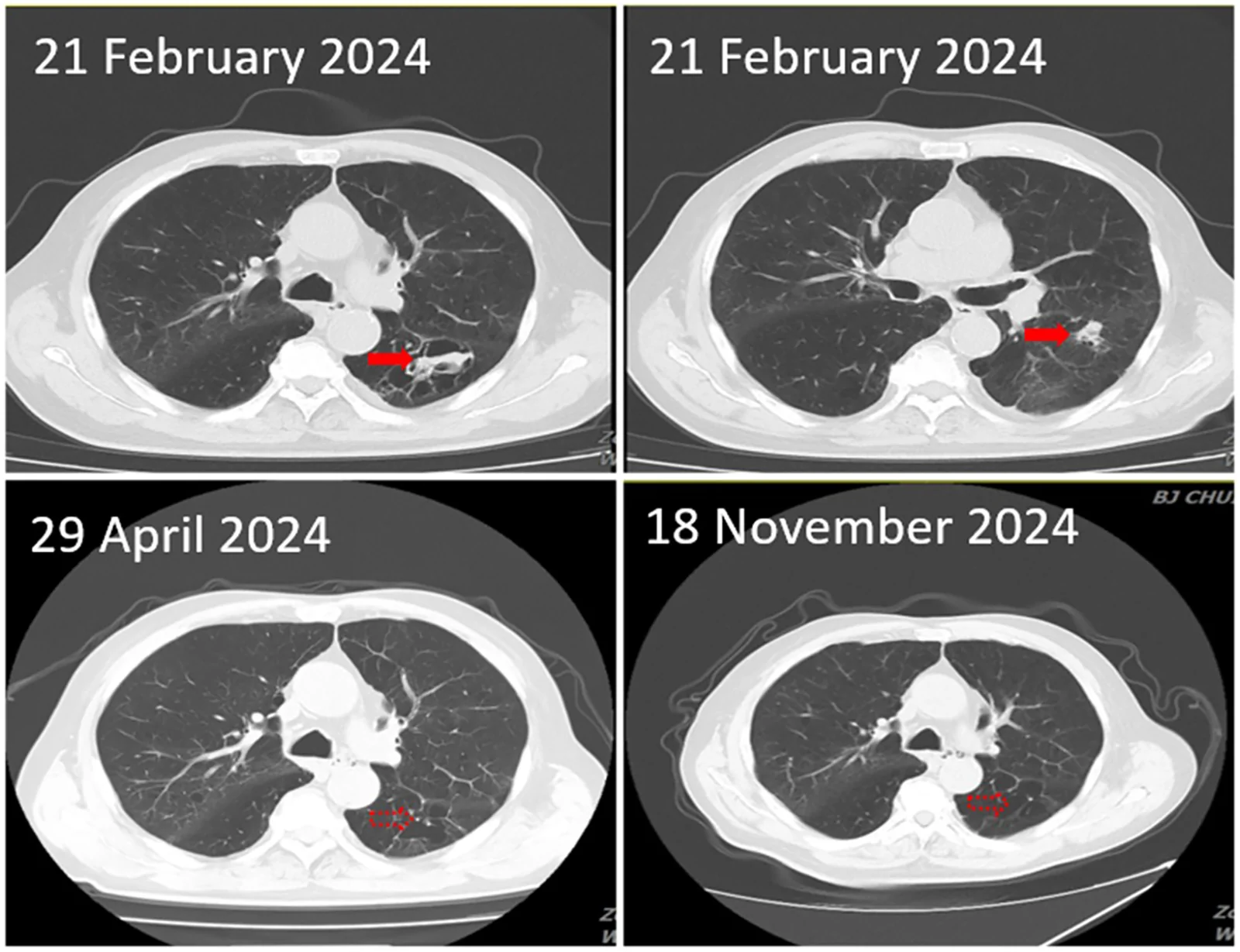
Chest CT before and after bronchial artery embolization in 2024. Solid red arrows indicate active pulmonary nodules; dashed red arrows indicate resolved lesions.

In May 2025, the patient experienced new episode of hemoptysis. Chest CT revealed a new cavitary nodule in the right middle lobe. Bronchoscopy showed a large amount of thick, dark red secretions in the right intermediate bronchus, with bloodstains observed in the right middle lobe after suctioning. BALF cultures were again negative for pathogens, and the galactomannan test was negative. The hemoptysis ceased after symptomatic treatment, and a follow-up chest CT demonstrated resolution of the pulmonary nodule (Figure 4).

**Figure 4:**
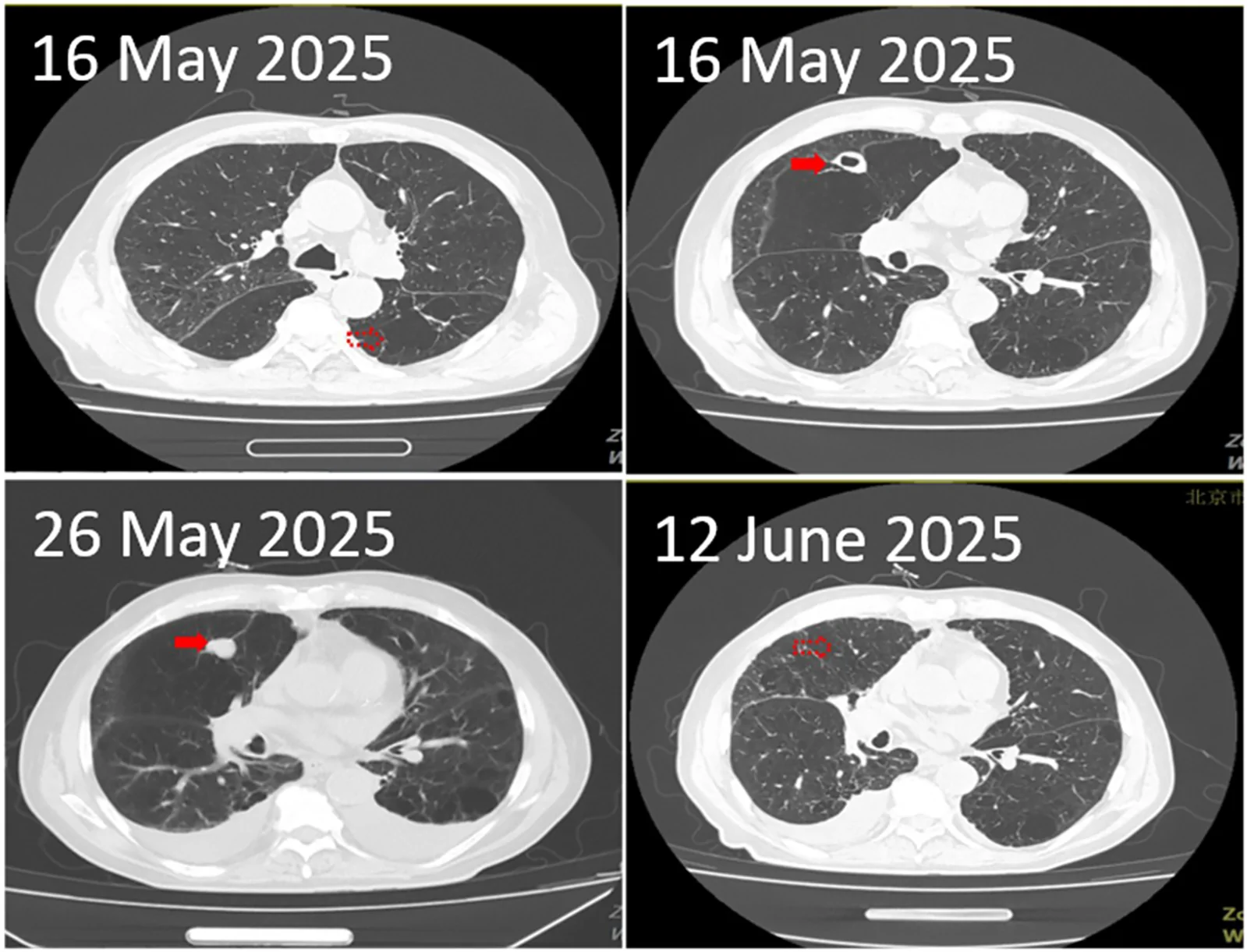
Chest CT findings during the 2025 episode. Solid red arrows indicate active pulmonary nodules; dashed red arrows indicate resolved lesions.

The patient had a 50-year smoking history of 20 cigarettes per day and had quit smoking 16 years prior. The patient has a 60-year history of chronic bronchitis, over 20 years of old pulmonary tuberculosis, more than 15 years of COPD, and a history of cerebral aneurysm rupture with hemorrhage 6 years ago. One year prior to admission, he was found to have mild anemia and mild renal insufficiency. Importantly, he has a 20-year history of polycystic kidney disease and polycystic liver disease (Supplementary Figure S1). The patient's estimated glomerular filtration rate (eGFR) fluctuated around 60 mL/(min·1.73 m^2^) for a long time. It had been persistently below 60 mL/(min·1.73 m^2^) since 2024 and decreased to 36 mL/(min·1.73 m^2^) during his most recent hospitalization. Subsequent peripheral blood genetic testing (Illumina Novaseq6000 platform, KingMed Diagnostics, Guangzhou, China) revealed that the patient carries a c.8426C > T (p.Pro2809Leu) variant, a missense mutation in the coding region of the Polycystin-1 (PKD1) gene.

All serial chest CT scans were acquired with 1-mm collimation and 1-mm thin-slice reconstruction, interpreted with standard lung and mediastinal window settings. Migratory pulmonary nodules were defined as transient nodular opacities (≤3 cm in maximal axial diameter) newly appearing in a different pulmonary segment/lobe compared with prior CT scans, with complete or near-complete resolution within 2–8 weeks after supportive-hemostatic therapy. DSA of bronchial arteries was performed via the transfemoral approach. Bronchial artery malformation was diagnosed by arterial dilatation (vessel diameter ≥2 mm) and marked tortuosity-meandering of the main trunk and branches.

## Discussion

Common causes of hemoptysis include pulmonary tuberculosis, acute respiratory tract infections, COPD, cancer, and bronchiectasis (7). Active pulmonary tuberculosis was excluded by negative BALF microbiology and absence of typical tuberculous lesions despite a history of old pulmonary tuberculosis. Acute infection-related hemoptysis was ruled out given absent infectious symptoms and repeatedly negative BALF cultures. Furthermore, the lesions resolved rapidly with only hemostatic therapy, without anti-inflammatory or immunosuppressive agents required for non-infectious inflammatory disorders. Neoplasm was excluded by complete resolution of pulmonary nodules on serial thin-section CT and absence of endobronchial masses at bronchoscopy. Hemoptysis in patients with COPD is generally limited to blood-tinged sputum unless complicated by severe bronchiectasis, which was not present in this patient. Thin-section CT showed no bronchial dilatation or wall thickening, ruling out bronchiectasis-related bleeding. Combined with the DSA findings during treatment, the patient's hemoptysis was highly likely associated with bronchial artery malformations. In cases of hemoptysis requiring intervention, 90% of bleeding originates from the bronchial arteries (7, 8).

This patient concurrently presented with hepatic cysts, intracranial aneurysm, and COPD, which are consistent with the common extrarenal features of ADPKD. Genetic testing revealed a c.8426C > T (p.Pro2809Leu) variant, indicating a diagnosis of ADPKD. Although this variant has not been reported in the large-scale population frequency database gnomAD, it has been documented in patients with polycystic kidney disease in the literature (9, 10). In reports of ADPKD patients with vascular complications (primarily dissections and aneurysms), almost all major arteries have been involved, including the aorta, coronary arteries, carotid arteries, vertebral arteries, and intracranial arteries (4). The mechanism underlying vascular complications in ADPKD is thought to be associated with Polycystin. Studies in mouse models have confirmed that PKD1 is highly expressed throughout the embryonic and adult cardiovascular system, including in the heart, aorta, and all major vessels (11). Polycystin is expressed in endothelial cells and vascular smooth muscle cells (VSMCs) that constitute the vessel wall. Deficiency of PKD1 in VSMCs can lead to progressive degeneration of elastic fibers in the arteries, resulting in vascular complications (12). Therefore, involvement of the bronchial arteries is also theoretically supported. Chronic inflammation from old pulmonary tuberculosis and persistent infection may theoretically cause bronchial-artery hypertrophy and tortuosity. However, the patient's tuberculosis was cured 20 years earlier, with no persistent infectious foci on CT. Thus, while post-tuberculous vascular remodeling cannot be fully excluded, we tend to favour a contributing role of ADPKD-associated vasculopathy in these bronchial-artery abnormalities. Furthermore, ADPKD may indirectly raise pneumothorax risk by increasing susceptibility to COPD, yet direct causal evidence linking ADPKD itself to spontaneous pneumothorax remains uncertain.

Although polycystin-1 is widely expressed in vascular smooth-muscle and endothelial cells throughout the systemic circulation, ADPKD-associated vascular complications are clinically dominated by lesions within aortic and intracranial arteries, raising an ongoing debate over whether polycystin-1 deficiency produces heterogeneous damage across distinct arterial beds (4). This uneven clinical phenotype may arise from regional differences in hemodynamic stress and intrinsic vessel-wall characteristics, though mutation-specific effects or modifier-gene influences cannot be excluded as contributing factors (13). Our case illustrates that bronchial arteries may also develop structural malformations in the setting of a PKD1 variant. Despite theoretical susceptibility conferred by systemic polycystin-1 dysfunction, bronchial-artery involvement remains scarcely documented in clinical practice, possibly due to under-recognition rather than genuine resistance to injury. Therefore, polycystin-1-mediated vascular harm is not biologically restricted to intracranial and aortic territories, yet the drivers of marked inter-bed phenotypic heterogeneity in human ADPKD remain incompletely resolved.

The limitation should be acknowledged. This is a single-case report without independent validation cohorts. Accordingly, definitive causal associations between PKD1 variation and bronchial artery malformation cannot be established, and additional clinical observations are needed to verify our findings.

## Conclusion

In conclusion, we report a single ADPKD patient presenting with concurrent bronchial artery malformation, recurrent hemoptysis and migratory pulmonary nodules. This observation raises the possibility that bronchial artery lesions might be an under-recognized vascular feature associated with ADPKD; further clinical data from additional patients are required to establish whether it constitutes a true complication of this disease.

## Data Availability

The original contributions presented in the study are included in the article/Supplementary Material, further inquiries can be directed to the corresponding author.

## Supplementary material

The Supplementary Material for this article can be found online at: https://www.frontiersin.org/articles/10.3389/fmed.2026.1949035/full#supplementary-material

Supplementary Figure S1 Abdominal imaging of autosomal dominant polycystic kidney disease. Solid red arrows indicate multiple cysts in both kidneys and the liver.
